# Kinetic-roughening diagrams between Kardar–Parisi–Zhang–like and Berezinskii–Kosterlitz–Thouless rough surfaces for steady crystal growth

**DOI:** 10.1038/s41598-025-18250-0

**Published:** 2025-10-03

**Authors:** Noriko Akutsu, Yoshihiro Kangawa

**Affiliations:** Research Institute for Applied Mechanics, Kyushu University, 6-1 Kasuga-koen, Kasuga, Fukuoka 816-8580 Japan

**Keywords:** Surfaces, interfaces and thin films, Statistical physics, thermodynamics and nonlinear dynamics, Computational science

## Abstract

Surface roughness at the nanometer scale influences various phenomena such as the morphology of crystallites at the micrometer scale, the structure factor in X-ray scattering, and backscattering in lidar used for passive remote sensing of ice clouds. In this study, the surface roughness and its roughness exponent are calculated for nano-scale two-dimensional surfaces in three dimensions during the steady growth of crystals using the Monte Carlo method on a lattice model. By monitoring the roughness exponent, slope-dependent kinetic roughening diagrams are constructed for universality classes. The diagrams provide insight into the intricate relationship between the roughness exponent and surface slope, as well as the driving force for crystal growth. The resulting diagrams reveal that at low temperatures, two Kardar–Parisi–Zhang (KPZ)-like kinetic roughening regions, KPZ-like1 and KPZ-like2, are separated by a region of Berezinskii–Kosterlitz–Thouless (BKT) roughening. At high temperatures close to but below the thermal roughening transition temperature of the (001) surface, another KPZ-like region, KPZ-like3, emerges for large driving forces for crystal growth. The terrace width histogram and surface height difference distribution function were also calculated using the Monte Carlo method and provide insight into how the surface crosses over to other classes or subclasses.

## Introduction

Surface roughness affects surface phenomena in many ways. A fluctuating surface is considered to obey the Family–Vicsek scaling function at non-equilibrium^1,2^ as $W(L,t)= L^{\alpha } f(t/L^z), \quad z=\alpha /\beta$, where *W*(*L*, *t*) is the standard deviation of the surface height at time *t* for a linear system size *L*, *f*(*x*) is a scaling function which converges to 1 for $x \rightarrow \infty$ and converges to $x^\beta$ for $x \rightarrow 0$, and $\alpha$, $\beta$, and *z* are the roughness, growth, and dynamic exponents. For steady crystal growth, $W(L)\propto L^{\alpha }$ for $\ t \rightarrow \infty$, and the surface width can be characterised by the roughness exponent $\alpha$ for a two-dimensional (2D) surface in three dimensions (3D). From a symmetry principle theory, the Kardar–Parisi–Zhang (KPZ) equation^3^ was proposed for growing surfaces. The exponents for the 2D KPZ surface in 3D are known numerically^4^.

**Fig. 1:**
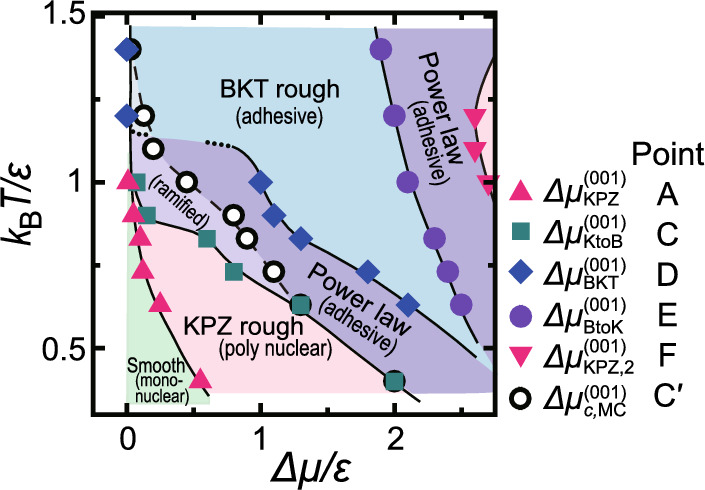
*T*–$\Delta \mu$ kinetic roughening diagram on the (001) surface^7^. *T* is temperature, $k_\textrm{B}$ is the Boltzmann constant, and $\epsilon$ is the microscopic ledge energy in the RSOS model (Eq. 3). $\Delta \mu$ is the driving force for crystal growth (Eq. 3), *i.e.* $\Delta \mu =0$ at the equilibrium, $\Delta \mu >0$: crystal growth, $\Delta \mu <0$: crystal retreat. The thermal roughening transition temperature of the (001) surface is $k_\textrm{B}T_\textrm{R}^{(001)}/\epsilon = 1.578$. Details are given in the “*T*–$\Delta \mu$ Kinetic roughening diagram” subsection in the “Results” section. The figure is taken from Ref. [7] under CC BY 4.0 licence.

It has been noted, however, that growing surfaces in crystal growth rarely exhibit KPZ behaviour^1,5^. Recently, Monte Carlo studies on a lattice model without surface or volume diffusion (interface-limited growth) have shown that a steadily growing (001) surface in a 2D poly-nucleation process produces KPZ-like kinetic rough surfaces in a wide parameter range at sufficiently low temperatures^6,7^ and another distinct form of KPZ-like area for high-*T* and $\Delta \mu$ (Fig. 1). The KPZ-like kinetic rough surface on a (001) surface near equilibrium is atomically smooth^8,9^ and the surface steps are well-defined. At the same time, *W*(*L*) of the (001) surface diverges as *L* increases in the order of $L^\alpha$, which means the surface is thermodynamically rough^1,2,10^. When the temperature increases, the KPZ-like kinetic rough region near equilibrium shrinks in the *T*–$\Delta \mu$ space and the Berezinskii–Kosterlitz–Thouless (BKT) rough region widens, with $W^2(L) \propto \ln L$.

KPZ-like kinetic rough surfaces near equilibrium on a nanometre-scale (001) surface have been shown to affect the crystallite shape at the micrometre scale and can explain the shape transformation of a Si crystallite in liquid Si^11^. Since a KPZ kinetic rough surface eliminates the cusp singularity^6,11^ in the kinetic Wulff plot, which is a polar plot of the surface growth rate *V*, the crystal growth shape (CGS) for a crystallite can be calculated by continuous equations. The strong anisotropy in *V* originating from the lattice structure remains because the KPZ -like kinetic rough surface is atomically smooth. This explains why the CGS has an approximate polyhedral shape, associated with the crystal symmetry, even though the surface is rough.

**Fig. 2:**
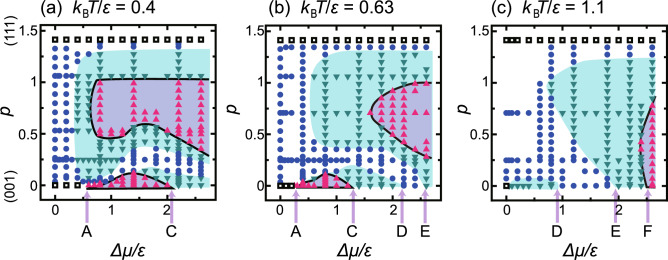
*p*–$\Delta \mu$ kinetic roughening diagrams. $p= \tan \theta$ where $\theta$ is the off angle of the tilted surface from the (001) surface towards the $\langle 111\rangle$ direction. $\Delta \mu$ is the driving force for crystal growth. Triangles: KPZ-like kinetic rough. Circles: BKT rough. Inverted triangles: Power-law rough. Open squares: Thermodynamically smooth. (**a**) $k_\textrm{B}T/\epsilon = 0.4.$ (**b**) $k_\textrm{B}T/\epsilon = 0.63.$ (**c**) $k_\textrm{B}T/\epsilon = 1.1.$ The shaded area represents the power-law kinetic rough region. The area enclosed by the black line is the KPZ-like kinetic rough region. Point A: $\Delta \mu _\textrm{KPZ}^{(001)}$. Point C: $\Delta \mu _\textrm{KtoB}^{(001)}$. Point D: $\Delta \mu _\textrm{BKT}^{(001)}$. Point E: $\Delta \mu _\textrm{BtoK}^{(001)}$. Point F: $\Delta \mu _\textrm{KPZ,2}^{(001)}$. The point designations follow Fig. 1. Details are given in the “*p*–$\Delta \mu$ Kinetic roughening diagram” subsection in the “Results” section.

**Fig. 3:**
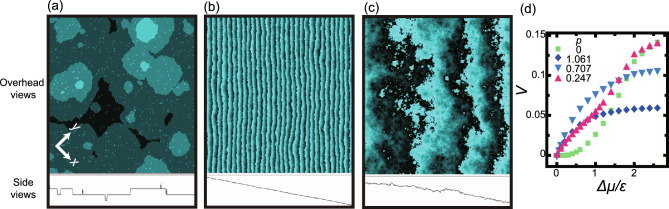
Examples of simulated KPZ kinetic rough surfaces and surface growth rate. $L= 320 \sqrt{2}$. (**a**), (**b**), and (**d**): $k_\textrm{B}T/\epsilon = 0.63$. (**c**): $k_\textrm{B}T/\epsilon = 1.1$. The shot time in (**a**), (**b**), and (**c**) is $4 \times 10^8$ MCS/site. The panel shows both an overhead and a side view. In the overhead views, brightness indicates the surface height, with the higher regions shown by brighter colours. So that the steps’ zigzag structure can be seen, the colour gradation is set in repeating groups of 10, such that the colour gradation returns to the lightest colour every 10 gradation steps as the height increases. (**a**) KPZ-like1. $\Delta \mu /\epsilon = 0.4$. $p=0$. (**b**) KPZ-like2. $\Delta \mu /\epsilon = 1.8$. $p=1/\sqrt{2} \approx 0.707$. $N_\textrm{step}= 320$. (**c**) KPZ-like3. $\Delta \mu /\epsilon = 2.6$. $p=32/(320 \sqrt{2}) \approx 0.0707$. (**d**) $\Delta \mu$ dependence of the surface steady growth rate *V*. The unit of *V* is $a/(\mathrm{MCS/site})$.

The sub-micron surface roughness is measured using X-ray scattering, where the structure factor $S(\vec {q})$ is expressed^12^ as $S(\vec {q}) =\frac{2\pi }{q_z^2} \int _0^\infty dR \ R e^{-q_z^2 g(R)/2} J_0(q_r R)$, where $\vec {q}$ is the wave vector transfer, $q_r = \sqrt{q_x^2+q_y^2}$, $J_0(x)$ is the zero-th order Bessel function, and *g*(*R*) is $\langle [z(X,Y)-z(0,0)]^2\rangle$ with $R= \sqrt{X^2+Y^2}$. For the self-affine surface at $q_r=0$ (crystal truncation rod (CTR) scattering^13^),

1 $$\begin{aligned} g(R) = A R^{2\alpha }, \ S(\vec {q}) \propto 1/q_z^{2+(2/\alpha )}, \end{aligned}$$

where *A* is the amplitude^12^. For a BKT rough surface similar to a liquid–gas interface,

2 $$\begin{aligned} g(R) = A' + B \ln R, \ S(\vec {q}) \propto \frac{2 \pi }{q_z^2} \textrm{e}^{- q_z^2 A'/2} \frac{2^{1-\eta }}{q_r^{2-\eta }}\frac{\Gamma (1-\eta /2)}{\Gamma (\eta /2)}, \end{aligned}$$

where $\eta =Bq_z^2/2$^12^. A similar diffraction pattern has been observed among optical diffraction patterns^14,15^.

Recently, the surface roughness and morphology of ice crystallites have drawn attention to the issue of estimating the backscattering, which is important for getting information with high precision from lidar (light detection and ranging) technology for the passive remote sensing of ice clouds^16,17^. Molecular dynamic simulations of ice by Mochizuki et al.^18^ suggested that the basal plane of a growing ice crystal exhibits a KPZ kinetic rough surface. Since the ice crystallites are spaceborne, the signals from various slopes of crystallites contribute to the lidar’s backscattering. When the surfaces in the sample are randomly oriented, such as for a powder, $S(\vec {q})$ should be averaged over the polar angle^12^ $\chi$ and $\phi$. If we designate the averaged $S(\vec {q})$ as $\tilde{S}(\vec {q})$, $\tilde{S}(\vec {q}) = [1/(4\pi )] \int \int S(\vec {q}) \sin \chi d \chi d \phi$. Thus, it can be seen that the slope dependence of surface roughness is necessary.

In this context, kinetic roughening for nano-scale “flat geometry”^19^ surfaces is studied in this article using the Monte Carlo method for the interface-limited steady growth of crystals. Surface diffusion^20^, dislocations^20^, and elastic effects^21–23^ on the surface are not taken into consideration. We focus on the effects of energy and entropy on the aggregation/decomposition of steps and the roughness of the (001) terrace. The goal of this study is to obtain *p*–$\Delta \mu$ kinetic roughening diagrams (Fig. 2) by monitoring the roughness exponent $\alpha$.

Note that our results are not directly obtained from solving the KPZ continuous equation^3^. The surface widths of surfaces under steady growth or retreat in a lattice model were calculated and classified by monitoring the roughness exponent $\alpha$. Hence, we denote surfaces with a KPZ value of $\alpha _\textrm{KPZ} = 0.3869$^4^ as “KPZ-like” kinetic rough surfaces. To investigate the physical ways a surface crosses over to a KPZ-like kinetic rough surface, we calculated the terrace width histogram (TWH) and the surface height difference distribution (SHD) of surfaces.

## Results

### Microscopic model

We adopted a restricted solid-on-solid (RSOS) model^24,25^ on a square lattice with nearest-neighbour (nn) height differences being restricted to zero or ± one. It should be noted that the height difference $\Delta h$ in the “RSOS model” in non-linear science research can take integer values, which corresponds to the absolute SOS (ASOS) model in surface roughening research^26^. The surface energy of a surface around a (001) surface, including (001) terrace roughness, is expressed by the following discrete Hamiltonian:

3 $$\begin{aligned} {\mathscr {H}}_\textrm{RSOS} = {\mathscr {N}}\epsilon _\textrm{surf}+ \sum _{n,m} \epsilon [ |h(n+1,m)-h(n,m)| +|h(n,m+1)-h(n,m)|] - \sum _{n,m} \Delta \mu \ h(n,m), \end{aligned}$$

where *h*(*n*, *m*) is the surface height at site (*n*, *m*), ${\mathscr {N}}$ is the total number of lattice points, $\epsilon _\textrm{surf}$ is the surface energy per unit cell on the planar (001) surface, and $\epsilon$ is the microscopic ledge energy for nn interactions. The summation with respect to (*n*, *m*) is taken over all sites on the square lattice. The RSOS condition is required implicitly. $\Delta \mu$ is the driving force for crystal growth. $\Delta \mu$ is defined by $\Delta \mu = \mu _\textrm{ambient}- \mu _\textrm{crystal}$, where $\mu _\textrm{ambient}$ and $\mu _\textrm{crystal}$ are the chemical potentials of the ambient phase and the crystal, respectively. Though $\epsilon _\textrm{surf}$ and $\epsilon$ depend on temperature because they correspond to the surface free energy in the atomic model^27^, we assume they are constant throughout this work.

The RSOS model can be mapped to a 2D XY spin model^28^, the 1D quantum spin 1 chain model^29^, and the 1D delta function Bose gas model^30^ at equilibrium. Unfortunately, the model cannot be solved exactly using the Bethe ansatz^31^. Therefore, the Monte Carlo method is adopted so that the entropy effect is precisely taken into account.

We studied our sample surface using the Monte Carlo method with the Metropolis algorithm for a non-conserved system with respect to the number of crystal atoms. The transition probability was 1 for $\Delta E= E_f-E_i \le 0$ and $\exp (-\Delta E/k_\textrm{B}T)$ for $0<\Delta E$, where $E_i$ is the surface energy of the initial configuration and $E_f$ is the surface energy of the proposed configuration. The surface energy was calculated using Eq. (3). Examples of simulated surfaces are shown in Fig. 3a–c. Since both RSOS and the Metropolis algorithm^32,33^ are well-established models, it is easy to determine whether the results are intrinsic to the model or an artefact with errors when an unexpected result is obtained. Our results for surface growth rate and the morphology of the RSOS model with the Metropolis algorithm agree well with the van Veenendaal et al. results^34^ obtained by the Monte Carlo method with the Kossel model with a kinetic Monte Carlo algorithm.

The first $2 \times 10^8$ Monte Carlo steps per site (MCS/site) are ignored. Then, the quantities are averaged over the following $2 \times 10^8$ MCS/site. The number of steps $N_\textrm{step}$ is fixed to give a surface slope of $p=N_\textrm{step}a/L$, where $a=1$ is the lattice constant. The surface growth velocity *V* is calculated as $V=(\langle h(t+\tilde{\tau })\rangle -\langle h(t)\rangle )/\tilde{\tau }$, where $\tilde{\tau }$ is set to be $2 \times 10^8$ MCS/site. In Fig. 3d, we show examples of simulated *V* for several surface slopes. The obtained *V* depends on *p* and $\Delta \mu$ in a reentrant way due to the competition between the 2D nucleation process on the (001) terraces and the step growth process.

For inclined surfaces, a squared surface width, which is a variance of the surface height, is calculated by

4 $$\begin{aligned} gW^2 =\langle \langle [h(\tilde{x}, \tilde{y}, t)- \langle h(\tilde{x}, t)\rangle _{\tilde{y}}]^2\rangle _{\tilde{y}} \rangle _{\tilde{x}}, \end{aligned}$$

where *W* is the surface width normal to the inclined surface, $g= 1+p_x^2 +p_y^2$ with $p_x=\partial \langle h \rangle /\partial x$ and $p_y=\partial \langle h \rangle /\partial y$ as geometrical factors, $\tilde{x}$ and $\tilde{y}$ are the [110] and $[ \bar{1}10 ]$ directions, respectively, and $\langle \cdot \rangle _{\tilde{y}}$ and $\langle \cdot \rangle _{\tilde{x}}$ are the averages over the $\tilde{y}$ and $\tilde{x}$ directions.

**Fig. 4:**
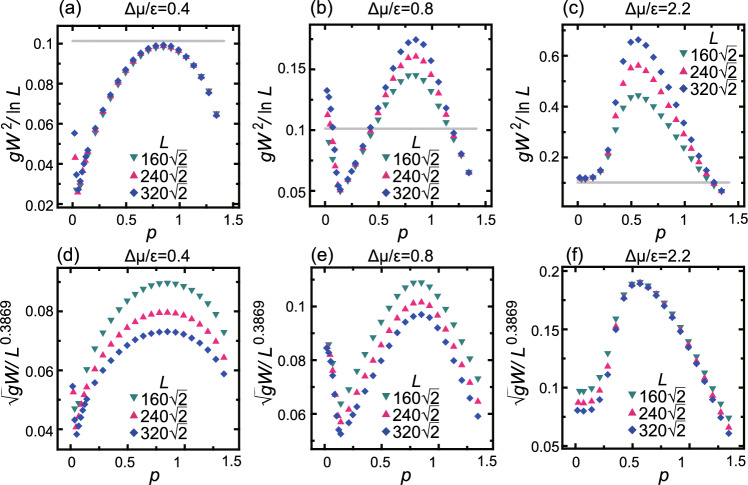
Slope dependence of the scaled surface width at several driving forces for crystal growth. $k_\textrm{B}T/\epsilon =0.63$. (**a**) and (**d**): $\Delta \mu /\epsilon =0.4$. (**b**) and (**e**): $\Delta \mu /\epsilon =0.8$. (**c**) and (**f**): $\Delta \mu /\epsilon =2.2$. (**a**), (**b**), and (**c**) are $gW^2$ scaled by $\ln L$. (**d**), (**e**), and (**f**) are $\sqrt{g}W$ scaled by $L^\alpha$, where $\alpha$ is assumed to be the value of KPZ obtained by Pagnani and Parisi^4^. Grey lines: $gW^2/\ln L= 1/\pi ^2 \approx 0.1013$, which is the BKT universal value at the thermal roughening transition point at equilibrium^36,37^.

An example of the slope dependence of the scaled surface width is shown in Fig. 4. Here, $gW^2$ is scaled by $\ln L$ in the upper panels, which assumes the surface is BKT rough. In the context of the KPZ equation, the Edwards–Wilkinson (EW) scaling seems to be appropriate^1,5^. We also plotted $gW^2$ vs. $\ln L$ and $(\ln L)^2$, where the roughness exponent is $\alpha =0$ for both. The $gW^2$ vs. $(\ln L)^2$ figure for $\Delta \mu /\epsilon =0.4$ shows poor linearity for $3<\ln (L/a) <7$ ($a=1$), while $gW^2$ v.s. $\ln L$ shows good linearity for $3<\ln (L/a) <7$^35^. In fact, in the area that is likely BKT rough in Fig. 4a, which is near equilibrium, the plotted data of different sizes overlap well. It has been well established that the thermal roughening transition at equilibrium belongs to the BKT universality class^10,36–43^ (see “Capillary waves create BKT rough surface” subsection in the “Method” section). As seen from Fig. 1, it is important to determine how the kinetic roughening is connected to the BKT thermal roughening, as discussed by Gilmer and Bennema^44^.

### *T*–$\Delta \mu$ kinetic roughening diagram

In our previous work^7^, the temperature *T*–$\Delta \mu$ kinetic roughening diagram for the (001) surface for steady growth was obtained. Fig. 1 is drawn based on the system size dependence^6,7^ of $gW^2/\ln L$ or $\sqrt{g}W/L^\alpha$, with $\alpha$ being assumed to be the KPZ value, $\alpha _\textrm{KPZ}=0.3869$^4^, similar to Fig. 4. If the plotted data for different sizes in the lower panels overlap well, we regard the points as having a roughness exponent $\alpha = \alpha _\textrm{KPZ}$, which means the points belong to the KPZ-like class. When the data do not overlap in either scaling, we denote them as “power-law” rough with $0<\alpha <\alpha _\textrm{KPZ}$. In the power-law rough region, $\alpha$ changes gradually depending on *p* and $\Delta \mu$. The characteristic points found on the (001) surface ($p=0$)^7^ are indicated on the right side of Fig. 1.

An area with $\alpha _\textrm{KPZ}$ is evident in a wide range of $\Delta \mu$ relatively near equilibrium at low temperatures next to the smooth surface area^6,7^. We call this the KPZ-like kinetic rough area. Since the surface width *W* increases as $L^\alpha$ as *L* increases, the surface is thermodynamically rough and the surface grows continuously in a 2D poly-nuclear growth mode. However, in contrast to the thermally roughened surface where the step free energy is zero, surface steps and terraces are apparent in the KPZ-like kinetic rough area, where the surface is atomically smooth. An example of the surface structure is shown in Fig. 3a. On the KPZ-like kinetic rough surface, island-on-island structures are frequently seen, creating a large length-scale period of surface undulations. In Ref. [7], the calculated data show a crossover point between 2D mono-nuclear growth area and 2D poly-nuclear growth area where finite values converge, depending on temperature; the converging values agree with the KPZ-like kinetic roughening transition point (label A in Fig. 1) within the numerical errors.

When the driving force for crystal growth $\Delta \mu$ is larger than $\Delta \mu _\textrm{KtoB}^{(001)}$ (label C in Fig. 1), no surface steps are discernible and the surface grows in an adhesive growth process. The surfaces for $\Delta \mu _\textrm{KtoB}^{(001)}<\Delta \mu$ are atomically and thermodynamically rough. The point C$'$, where the surface growth rate starts to increase linearly with $\Delta \mu$, is the “kinetic roughening point”, as usually defined in the crystal growth area.

At high temperatures, the BKT rough surface area expanded and became connected to the thermally roughened surface area, which is consistent with the results reported by Gimer and Bennema^44^, Van Veenendaal et al.^34^, and Cuppen et al.^45^. At high *T* and large $\Delta \mu$^7^, where the surface widths are large and the contour of the surface is ramified, another KPZ-like kinetic rough area was found, separated from the low temperature KPZ-like kinetic rough surface.

### *p*–$\Delta \mu$ kinetic roughening diagram

Figure 2 shows the *p*–$\Delta \mu$ kinetic roughening diagrams for several temperatures. Figure 2 is also drawn based on the system size dependence of $gW^2/\ln L$ or $\sqrt{g}W/L^\alpha$, with $\alpha$ being assumed to be $\alpha _\textrm{KPZ}$. Examples of the scaled surface width are shown in Fig. 4. The characteristic points found on the (001) surface ($p=0$, Fig. 1) are indicated on the horizontal axes by the letters A, C, $\cdots$, and F. As a crude criterion, the surface is likely to be BKT rough when $gW^2/\ln L < 1/\pi ^2$, where capillary waves are dominant.

From Fig. 2a and b, we can see: (i) The surfaces near equilibrium belong to the BKT class, as do the tilted surfaces at equilibrium^10,36–41^. (ii) Two KPZ-like or power-law kinetic rough areas are separated by the BKT rough area. (iii) One KPZ-like area lies around the (001) and its vicinal surface is between points A and C in Fig. 2 a and b (KPZ-like1). (iv) The KPZ-like areas are wide at low temperatures, whereas the areas shrink rapidly to zero as temperature increases. This result is consistent with the result obtained on the (001) surface (Fig. 1).

On the other hand, the (111) surface and terraces, which form an almost ideal terrace–step–kink (TSK) model, are atomically and thermodynamically smooth due to the RSOS condition. For $1/\sqrt{2} {\mathop {\sim }\limits ^{<}}p$, steps with (111) terraces are formed and become KPZ-like kinetic rough surfaces (Fig. 3b, KPZ-like2).

At high temperatures and at large $\Delta \mu$ for $p {\mathop {\sim }\limits ^{<}}1/\sqrt{2}$, as demonstrated in Fig. 3c, the KPZ-like kinetic rough surface is atomically and thermodynamically rough (KPZ-like3) and surface steps are no longer visible. This area also emerges in Fig. 1 at high *T* and large $\Delta \mu$ separated from the KPZ-like1 area.

### KPZ-like2 caused by step-meanderings

**Fig. 5:**
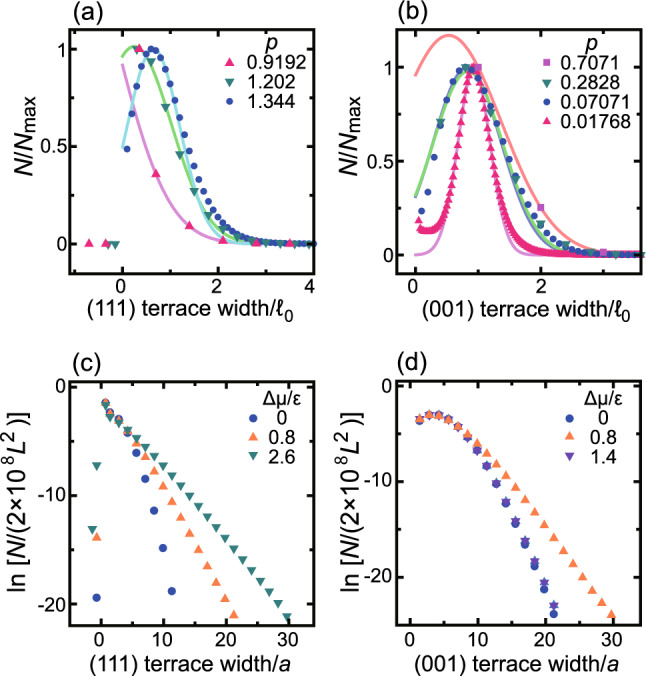
Histograms of terrace widths. $k_\textrm{B}T/\epsilon = 0.4$. $L=320 \sqrt{2}$. (**a**) Histogram of the (111) terrace width $\ell$ divided by $\ell _0$, the mean distance between adjacent steps. $\Delta \mu /\epsilon =0.8$. *N*: frequency of the terrace width. $N_\textrm{max}$: maximum value of N. (**b**) Histogram of the (001) terrace width divided by $\ell _0$. $\Delta \mu /\epsilon =0.8$. (**c**) Logarithmic histogram of the (111) terrace width. $p=(7\sqrt{2})/40 \approx 1.061$. (**d**) Logarithmic histogram of the (001) terrace width. $p=(3\sqrt{2})/4 \approx 0.247$.

The terrace width histograms (TWHs) of stepped surfaces calculated by the Monte Carlo method are shown in Fig. 5a and b, for steps with (111) and (001) terraces, respectively, illustrating the slope dependence of the histograms. Here, *N* is the frequency of the terrace width $\ell$, and $\ell _0$, which is a characteristic length for a tilted surface, is the mean terrace width defined by $\ell _0=a/(\sqrt{2}-p)$ and $\ell _0=a/p$ for steps with (111) and (001) terraces, respectively. The values of $\ell _0$ are listed in Table 1. The horizontal axes of Fig. 5a and b are scaled by $\ell _0$. For the steps with (111) terraces, the adatoms, holes, and their clusters on the (001) side surface of merged steps are regarded as “overhangs”. Hence, the terrace width is counted with negative values.

In contrast to a stepped surface^46–48^ with Calogero–Sutherland type step–step repulsion^21,22^, where $N|_{p=0}=0$ due to the step–step repulsion, the present results show a finite value of $N|_{p=0}$ because two steps can occupy one site for a tilted surface inclined towards the (111) surface. If we assume $\beta =2$ for the Tracy–Widom distribution, as thought to be the case with no step–step interactions, the terrace width distribution $P_{\beta } (\ell )$ must be $P_{\beta } (\ell ) \propto \ell ^{\beta } \exp [-A_{\beta } \ell ^2]$, where $A_{\beta }$ is a constant^5,48^. However, the TWHs shown in Fig. 5a and b disagree with this.

**Table 1:** Mean terrace width and its standard deviation $\sigma$ ($k_\textrm{B}T/\epsilon =0.4$, $\Delta \mu /\epsilon =0.8$).

*p*	$\ell _0/a$	$\ell _{N\mathrm max}/\ell _0$	$\sigma /\ell _0$	
(111) terrace				
0.7071	1.414	−2	1.1	KPZ-like2
0.9192	2.020	−1.2	1.1	KPZ-like2
1.202	4.714	0.2	0.8	power law
1.344	14.14	0.6	0.5	BKT
1.397	56.57	0.4	0.3	BKT
(001) terrace				
0.01768	56.57	0.9	0.3	KPZ-like1
0.07071	14.14	0.8	0.5	BKT
0.2828	3.536	0.8	0.6	power law
0.7071	1.414	0.5	0.8	KPZ-like2

EMPTY

In Fig. 5c and d, the $\Delta \mu$ dependencies of the logarithms of TWHs are shown for steps with (111) and (001) terraces, respectively. For the TWH for (111) terraces (Fig. 5c), the frequencies of the large width increase gradually as $\Delta \mu$ increases for the (111) terraces at $p=1.061$. The logarithm of the TWH for the (111) terraces decreases linearly for a large $\Delta \mu$, where the surface becomes power-law kinetic rough (Fig. 2a). We assume the shape of the TWH to be the product of Gaussian and exponential decay functions:

5 $$\begin{aligned} N/N_\textrm{max}= c_1 \exp [-(\ell /\ell _0-1)^2/\{2(\sigma /\ell _0)^2\} - c_2 \ell /\ell _0] \end{aligned}$$

where $N_\textrm{max}$ is the maximum value of *N*. Using the least square method, the Monte Carlo results are fitted to Eq. (5) around $\ell \sim \ell _0$ by adjusting $\sigma$, $c_1$, and $c_2$. The obtained lines are shown in Fig. 5a and b. The obtained $\sigma /\ell _0$ and $\ell _{N\mathrm max}/\ell _0$, where $\ell _{N\mathrm max}/\ell _0$ is the $\ell$ at the maximum value of N, are listed in Table 1.

When $c_2 \ne 0$, $\ell _{N\mathrm max}/\ell _0$ must shift from 1. For the (111) terraces, the calculated data for $\ell /\ell _0<10$ are well reproduced by the fitting lines. $\ell _{N \textrm{max}}/\ell _0$ decreases to negative values as the step density increases in Table 1. The values of $\sigma /\ell _0$ in Table 1 show that the surface becomes KPZ kinetic rough when $1 \lesssim \sigma /\ell _0$ (KPZ-like2). This large $\sigma /\ell _0$ is realized when $\ell _0$ becomes small, which indicates that the step meanderings become larger than $\ell _0$. When $\sigma /\ell _0 \le 0.5$, the TSK-like surface becomes BKT rough. This case is a capillary-wave^10,40,42^ dominant regime. The surface becomes power-law rough for $0.5 < \sigma /\ell _0 \lesssim 1$, forming a crossover region.

For the (001) terraces, however, the agreement between the Monte Carlo data and the best fit lines to Eq. (5) becomes poorer, especially away from $\ell /\ell _0=1$ (Fig. 5b). As $\Delta \mu$ increases at $p=0.247$, the frequencies with large widths show a reentrant behaviour (Fig. 5d). The only difference between the (001) and (111) terraces is the presence of thermally excited structures, such as adatoms, holes, islands, and clusters of holes. Hence, it is concluded that the reentrant behaviour is caused by such thermally excited structures on the (001) terraces. These structures enhance step advancement when they are at the same height level and inhibit step advancement when they are at different height levels due to the RSOS condition. The step advances as if it is moving across a plane with holes that change position over time. A plane with holes is not homeomorphic to a plane without holes. In this manner, the structures on the (001) terrace suppress the free meandering of steps, reducing the Gaussian meandering of steps perceived as capillary waves.

For the small *p* stepped surface with (001) terraces (Fig. 5b), the frequencies of the small widths less than $\ell /\ell _0 \approx 1$ are large relative to the Gaussian histogram due to the island-on-island structure on the (001) terraces. $\ell _{N \textrm{max}}/\ell _0$ are about one, except for the case of $p=0.7071$. This suggests that the KPZ-like kinetic rough (001) surface and its vicinal surfaces (KPZ-like1) have different characteristics from the KPZ-like kinetic rough surface with (111) terraces (KPZ-like2).

### BKT rough area separating the KPZ-like kinetic rough areas

**Fig. 6:**
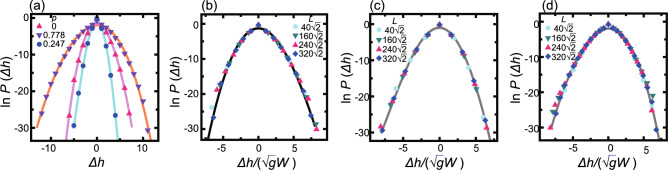
Logarithm of surface height difference distribution function. $k_\textrm{B}T/\epsilon = 0.4$. $\Delta \mu /\epsilon = 1.4$. $L=320 \sqrt{2}$. (**a**) $\ln P(\Delta h)$ v.s. $\Delta h$ for three values of *p*. (**b**), (**c**), and (**d**) $\ln P(\Delta h)$ vs. $\Delta h/(\sqrt{g}W)$ (**b**) $p=0$. KPZ-like1 kinetic rough surface. (c) $p=0.247$. BKT rough surface. (**d**) $p=0.778$. KPZ-like2 kinetic rough surface. Lines: Eq. (10) with the coefficients given in Table 3.

**Table 2:** Statistics for the surface height difference distribution functions ($L/a=320 \sqrt{2}$).

Class	$k_\textrm{B}T/\epsilon$	$\Delta \mu /\epsilon$	Surface slope *p*	$\sqrt{g}W/a$	Skewness	Excess kurtosis
KPZ-like1	0.4	1.4	0	0.944	0.048	− 1.33
KPZ-like1	0.4	− 1.4	0	0.944	− 0.048	− 1.33
KPZ-like1	0.63	0.8	0	0.907	0.046	− 1.39
KPZ-like2	0.4	1.4	0.778	1.626	− 0.103	− 0.65
KPZ-like2	0.4	− 1.4	0.778	1.634	0.101	− 0.64
KPZ-like2	0.63	1.6	0.707	1.491	− 0.109	− 0.72
KPZ-like3	1.1	2.6	0	1.996	− 0.149	− 0.41
KPZ-like3	1.1	2.6	0.354	2.08	− 0.132	− 0.40

EMPTY

To obtain information on the KPZ-like subclasses, the surface height difference distribution (SHD) functions $P(\Delta h)$ were calculated (Fig. 6) along with the statistical data for the SHD (Table 2). Here, $\Delta h$ was calculated as $\Delta h= h(i,j)-\langle h(i,,j) \rangle _{\tilde{y}}$. Details of the lines in Fig. 6 are given in the “Lines for Fig. 6” subsection in the “Method” section. Since the SHD data at $\Delta h =0$ are averted upward from the lines, we calculated the skewness and excess kurtosis by excluding the value of SHD at $\Delta h =0$.

At the (001) surface ($p=0$, KPZ-like1), the SHD has a positive skewness, whereas for the inclined surfaces between the (001) and (111) surfaces, the SHD has negative skewness (KPZ-like2). The sign change of the skewness as the surface slope *p* increases explains the appearance of the BKT rough surface. When the value of the skewness decreases to zero as *p* increases, the Gaussian SHD makes the surface BKT rough^37,43^ as explained in the “Capillary waves create BKT rough surface” subsection in the “Methods” section. In this manner, the BKT rough area separates the KPZ-like1 and KPZ-like2 areas. Example values of $\sqrt{g}W$, skewness, and excess kurtosis for the BKT rough region separating the two KPZ-like regions are 0.635, 0.042, and $-1.90$, respectively, for $p=0.2475$, $k_\textrm{B}T/\epsilon = 0.4$, and $\Delta \mu /\epsilon = 1.4$. The fourth order term in the right hand side in Eq. (10) for the line in Fig. 6c makes the tails of $P(\Delta h)$ with $|\Delta h|$ large, which leads to small kurtosis.

The reason the SHD for the (001) surface (KPZ-like1) has a positive skewness is that an island-on-island structure created by the 2D poly-nuclear process has a bell-shaped protrusion (Fig. 3a). The step on the top height level advances freely until the step catches up with the step below. The advancement of the step is partially stalled by the step below due to the RSOS condition. Hence, the population of positive $\Delta h$ becomes larger than that of negative $\Delta h$. For a stepped surface (KPZ-like2), meanderings of steps create concave surface undulations. The step on the lowest level advances freely. However, the step on the level above is partially stalled due to the RSOS condition. This suppression of step advancement becomes stronger for the step on the higher level. This leads a larger population of negative $\Delta h$ than positive $\Delta h$.

#### Crystal retreat

In the case of crystal retreat such as evaporation, dissolution, or melting, the kinetic roughening diagrams are considered to be the same, replacing $\Delta \mu$ with $|\Delta \mu |$. We confirmed that a *V*–$\Delta \mu$ plot, such as Fig. 3d, is symmetric when replacing *V* and $\Delta \mu$ with $-V$ and $-\Delta \mu$ for $\Delta \mu /\epsilon = -1.4$, $k_\textrm{B}T/\epsilon = 0.4$, and $p=0$ and 0.778 . We also confirmed this symmetry in Ref. [35]. Since the RSOS Hamiltonian Eq.(3) is symmetric for the replacement of *h*(*n*, *m*) and $\Delta \mu$ with $- h(n,m)$ and $- \Delta \mu$, the observed symmetry in *V* and $\sqrt{g}W$ is consistent with the expectations.

In crystal retreat, the hole-in-hole structures created by the 2D poly-nuclear process favour cup-shaped surface undulations, and a receding step is partially stalled by a step on the level above due to the RSOS condition. For a stepped surface, meanderings of steps have convex surface undulations. Therefore, the (001) surface (KPZ-like1) has a negative skewness, whereas a stepped surface (KPZ-like2) has a positive skewness. The absolute values of $\sqrt{g}W$, skewness, and kurtosis at $\Delta \mu /\epsilon =-1.4$ are the same as the ones at $\Delta \mu /\epsilon = 1.4$ within the numerical error (Table 2).

### Subclasses in the KPZ-like class

The calculated statistical data of SHD indicate that three subclasses in the KPZ-like kinetic rough surface exist. It should be noted that the values of the statistical data are not the same as those of the usual (2+1)-dimensional KPZ system^19,49^. In Halpin-Healy’s work^49^, the values of skewness and kurtosis for the (2+1)-dimensional KPZ system are given as 0.427 and 0.349, respectively. The usual KPZ area is thought to exist far from equilibrium. However, the KPZ-like1 and KPZ-like2 areas exist near equilibrium, as shown in Figs. 1 and 2a and b. Therefore, from the data, a system with KPZ-like subclasses is considered to be different from the usual (2+1) KPZ classes. As stated at the end of the introduction, the present results are not based on direct calculations of the solution of the continuous KPZ equation^3^ for 2D surface. It is therefore surprising that the numerical results in this work for the roughness exponent for steady growth/retreat have the same values of the KPZ at relatively near equilibrium.

The KPZ-like region at $k_\textrm{B}T/\epsilon = 1.1$ for large $\Delta \mu$, which was designated KPZ-like3, shows different statistical data from the KPZ-like1 or KPZ-like2 regions (Table 2). The surface steps are no longer discernible, since the surface is atomically and thermodynamically rough (Fig. 3c). The values of $\sqrt{g}W$ in the KPZ-like3 region are larger than those in the KPZ-like1 and KPZ-like2 regions, and thus the surface in the KPZ-like3 region is considered to be relatively close to the (2+1) interface of the solution of the original KPZ equation. However, the values of skewness and kurtosis of the SHD differ from those of KPZ.

The characteristic of a KPZ-like1 or KPZ-like2 kinetic rough surface is that the surface undulations are created by many surface steps. This is the central idea of the TSK model. The relationship between a surface width and a step width based on the TSK model are given in Refs.^39–42^ at equilibrium. On the stepped surface at equilibrium, the roughness exponent of a single step becomes zero due to entropic step-step interactions. A step on a KPZ-like1 kinetic rough surface is circular on average and a step on a KPZ-like2 kinetic rough surface is straight on average (Fig. 3a and b). A single surface step can be regarded as a 1D KPZ interface. The histogram of the rescaled local height for the circular and linear (1+1) interfaces show the shapes relating to a random matrix of Gaussian unitary ensembles (GUE) for a circular interface and Gaussian orthogonal ensembles (GOE) for a flat interface, respectively^50,51^. The KPZ-like1 kinetic rough surface consists of many circular steps and the KPZ-like2 kinetic rough surface consists of many strait steps, on the whole. The many-body system of 1D KPZ steps in the KPZ-like1 or KPZ-like2 regions would not be a system with the solution of the original KPZ continuous equation^3^. Entropic step-step interactions may change step’s properties. However, the 2D surfaces of the KPZ-like subclasses inherit the step (1D interface) subclasses (Figs. 1 and 2).

The most crucial difference between the well-known KPZ systems and the KPZ-like system considered in this study is whether it exhibits steady growth. The only exponent available in steady growth is the roughness exponent $\alpha$. Hence, we use $\alpha$ to classify the kinetic roughening phenomena. KPZ-like attractors, which are like a fixed point at critical phenomena at equilibrium, attract systems in the long time limit. From the SHD data, we consider that KPZ-like attractors are different from the KPZ attractor. However, in that case, we need to explain why KPZ-like attractors have the same roughness exponent as the KPZ attractor, for which we do not yet have an explanation. This is a problem to be clarified in a future study.

## Conclusions

*p*–$\Delta \mu$ kinetic roughening diagrams are constructed for inclined surfaces between the (001) and (111) surfaces for steady crystal growth/retreat by monitoring the roughness exponent $\alpha$ using the Monte Carlo method based on the RSOS model with $\Delta h = \{0, \pm 1\}$.

- The *p*-$\Delta \mu$ kinetic roughening diagram exhibits KPZ-like, power law, and BKT areas, where the roughness exponents are $\alpha = \alpha _\textrm{KPZ}=0.3869$, $0<\alpha <\alpha _\textrm{KPZ}$, and $\alpha = 0$, respectively.
- There are three KPZ-like subclasses for inclined surfaces between the (001) and (111) surfaces: KPZ-like1, KPZ-like2, and KPZ-like3. The KPZ-like subclasses inherit the KPZ subclass of steps (one-dimensional interfaces).
- Surfaces close to the equilibrium state belong to the BKT class, including inclined surfaces at equilibrium. For the area with $\alpha =0$, $gW^2= A(p,T,\Delta \mu ) \ln L$ (BKT) is a better fit for the calculated data than $\sqrt{g}W= A'(p,T,\Delta \mu ) \ln L$ (EW).
- The KPZ-like1 area lies around the (001) and its vicinal surface is between points A and C in Figs. 1 and 2a and b at low temperatures. The KPZ-like1 kinetic rough surface is atomically smooth but thermodynamically rough. The surface grows in a 2D poly-nucleation process. Island-on-island structures or multi-body circular steps lead to a KPZ-like1 kinetic rough surface (Fig. 3 a).
- A surface with a train of steps, which is also atomically smooth and thermodynamically rough, belongs to the KPZ-like2 kinetic rough class, when step meanderings are well developed (Fig. 3b, Table 1). The terrace width histogram can be approximately expressed by a truncated Gaussian when the surface is BKT rough. For a KPZ-like2 kinetic rough surface, the histogram shows an approximately exponential decay.
- A BKT rough area separates the KPZ-like1 and KPZ-like2 areas. The skewness of the distribution function of the surface height difference (SHD) is determined by the local morphology of the surface undulation, with a bell-shape for positive skewness and cup-shape for negative skewness. The skewness of the SHD distribution function for a KPZ-like1 class surface has a different sign from that for the KPZ-like2 class due to the RSOS condition. As *p* increases from 0 to $\sqrt{2}$, a positive (negative) skewness of the SHD distribution function gradually decreases (increases) to negative (positive) values for crystal growth (retreat). At a point in this process, the surface will have zero skewness, resulting in a BKT rough surface.
- As the temperature increases, the KPZ-like1 and KPZ-like2 areas shrink as small clusters of adatoms and holes form on (001) terraces by thermal fluctuations, hindering free step fluctuations and decrease $\alpha$. However, at high temperatures and large $\Delta \mu$, the surface becomes a KPZ-like3 kinetic rough surface (Fig. 3c).

## Methods

### Capillary waves create BKT rough surface

After expanding $h(\vec {x})$ into a Fourier series such as $h(\vec {x}) = \Sigma _k h(\vec {k}) \exp (i \vec {k}\cdot \vec {x})$, where $i^2=-1$, $\vec {k}=(k_1,k_2)$, $k_j=(2\pi m_j )/L$ $(j=1,2; m_j=\pm 1, \pm 2, \cdots )$, the squared surface width can be expressed as

6 $$\begin{aligned} W(\vec {n})^2=\langle [h(\vec {x})-\langle h(\vec {x}) \rangle ]^2 \rangle /g= \Sigma _k \ \langle h(\vec {k}) h(-\vec {k})\rangle /g = \frac{k_\textrm{B}T}{gL^2} \ \Sigma _k \ [\Sigma _{\alpha , \beta } \ f^{(2) \alpha \beta }]^{-1}, \end{aligned}$$

where $\vec {n}$ is a unit vector normal to the surface and $\langle \cdot \rangle$ is an average over the capillary wave Hamiltonian

7 $$\begin{aligned} {\mathscr {H}}_{cw} = \frac{1}{2} \Sigma _{i,j} \int _0^L \textrm{d}x^1 \int _0^L \textrm{d}x^2 f^{(2) ij}(\vec {p_e})\cdot \Delta p_i(\vec {x})\cdot \Delta p_j(\vec {x}), \quad \Delta \vec {p}(\vec {x}) = \vec {p} (\vec {x}) - \vec {p_e}, \quad \vec {p}(\vec {x})= \left( \frac{\partial h(\vec {x})}{\partial x^1}, \frac{\partial h(\vec {x})}{\partial x^2} \right) , \end{aligned}$$

where $f^{(2) ij}(\vec {p_e})$ is the surface stiffness tensor at the mean surface gradient $\vec {p_e}$. The capillary wave Hamiltonian is derived from the generalised free energy for the inclined surface^37^. The right hand side of Eq. (6) can be evaluated using the standard continuous approximation and a change of variables:

8 $$\begin{aligned} W(\vec {n})^2=\frac{k_\textrm{B}T}{2\pi g\sqrt{\det f^{(2)} } } \int _\Lambda ^u \textrm{d}q \ q^{-1}, \quad u=1/a, \quad \Lambda = \textrm{const} \times (1/L), \quad \textrm{as} \ L \rightarrow \infty . \end{aligned}$$

Thus, we have

9 $$\begin{aligned} W(\vec {n})^2 \approx \frac{k_\textrm{B}T}{2\pi g\sqrt{\det f^{(2)} } } \ \ln L, \quad \textrm{as} \ L \rightarrow \infty . \end{aligned}$$

At the thermal roughening transition temperature $T_\textrm{R}^{(001)}$ at equilibrium, we have $W(\vec {n})^2=(1/\pi ^2) \ln L$, where $1/\pi ^2$ relates to the BKT universal value $2/\pi$, from the BKT results of Jayaprakash et al.^36^ and the relationship between the surface stiffness tensor and curvature tensor^37^.

### Lines for Fig. 6

The surface height difference distribution (SHD) functions $P(\Delta h)$ were calculated and are shown in Fig. 6. The lines for Fig. 6a–d are given by

10 $$\begin{aligned} \ln P(x)= a+bx+ cx^2 +dx^3+ex^4, \end{aligned}$$

where $x= \Delta h/(\sqrt{g}W)$. The respective values of the coefficients are listed in Table 3. The sign of *d* is positive for the KPZ-like1 class and negative for the KPZ-like2 class.

**Table 3:** Parameters of lines in Fig. 6. $k_\textrm{B}T/\epsilon =0.4$. $\Delta \mu /\epsilon =1.4$.

Class	Surface slope *p*	*a*	*b*	*c*	*d*	*e*
KPZ-like1	0	− 1.50	− 0.0490	− 0.494	0.0142	− 0.000814
BKT	0.247	− 1.00	− 0.0409	− 0.632	− 0.0105	0.00141
KPZ-like2	0.778	− 1.95	0.0260	− 0.474	− 0.0128	− 0.000931

## Data Availability

The datasets used and/or analysed in the current study are available from the corresponding author upon reasonable request.
